# Primary Synovial Sarcoma of the Mediastinum : A Case Report

**DOI:** 10.1155/2011/602853

**Published:** 2011-09-07

**Authors:** Gayatri Ravikumar, Shalini Mullick, Anuradha Ananthamurthy, Marjorie Correa

**Affiliations:** Department of Pathology, St. John's Medical College, Sarjapur Road, Koramangala, Bangalore 560034, India

## Abstract

Synovial sarcomas commonly occur in the extremities of young adults. A primary occurrence in the mediastinum is very rare with only a few reported cases in the world literature. This paper is about a 42-year-old male who presented with chest pain and dyspnoea on exertion. Imaging showed an anterior mediastinal mass with adhesions to the lung. Pathological examination of the resected mass showed a biphasic neoplasm with a spindle cell component admixed with gland-like elements. The tumour showed positive staining with cytokeratin, epithelial membrane antigen, and Bcl-2 confirming the diagnosis of a biphasic synovial sarcoma. A wide range of neoplasms, both primary and metastatic, occur in the mediastinum, which pose considerable diagnostic difficulties. A synovial sarcoma should always be considered in the differential diagnosis, and immunohistochemistry is an important adjuvant tool in this situation. This paper highlights the importance of recognizing an unusual presentation of this aggressive neoplasm to aid appropriate clinical management.

## 1. Introduction

Synovial sarcoma is a distinctive malignant soft tissue neoplasm that commonly occurs in the extremities of young adults. Although frequently associated with joints, tendons and bursal structures, it is now believed that this tumour does not originate from synovial cells as originally postulated. In fact, these tumours show epithelial differentiation and are thought to be derived from pluripotent mesenchymal cells capable of epithelial differentiation [1]. 

Uncommonly, synovial sarcomas have been reported from rare sites such as head and neck region, abdomen, thoracic cavity, and mediastinum. We have described here a case of biphasic synovial sarcoma occurring primarily in the mediastinum. The mediastinum is a rare site for occurrence of a primary synovial sarcoma with only a few case reports and series published in the literature till date. A wide range of tumours occur in the mediastinum, and the differential diagnosis of these tumours can be quite challenging. 

## 2. Case Report

**Figure 1:**
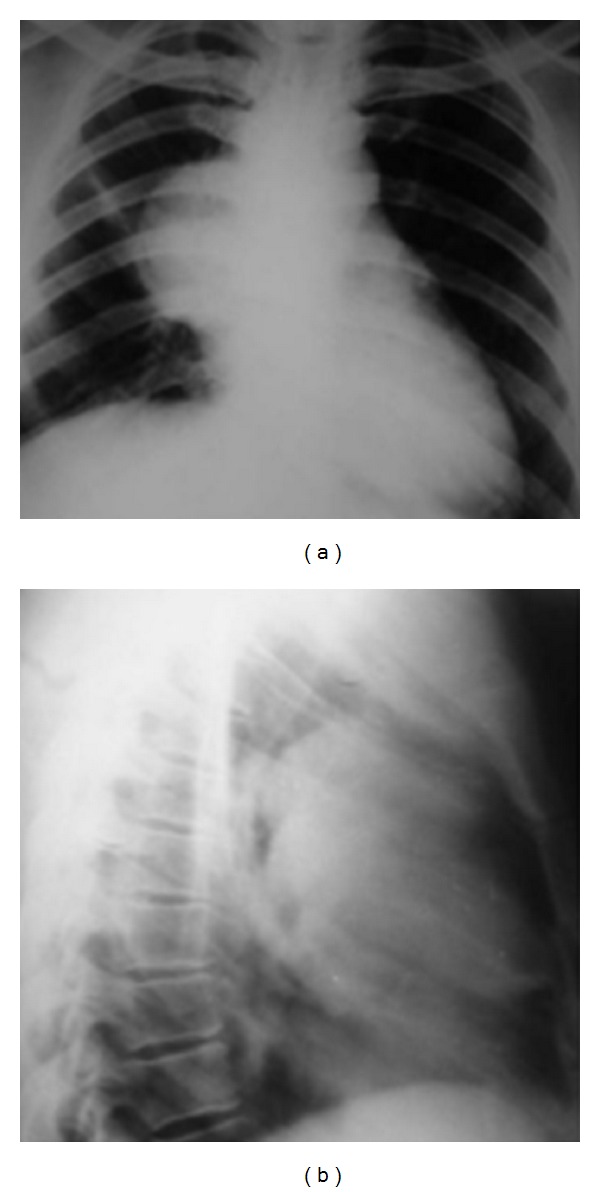
Chest radiographs, PA (a) and lateral views (b) show a well-defined mass in the anterior mediastinum.

**Figure 2:**
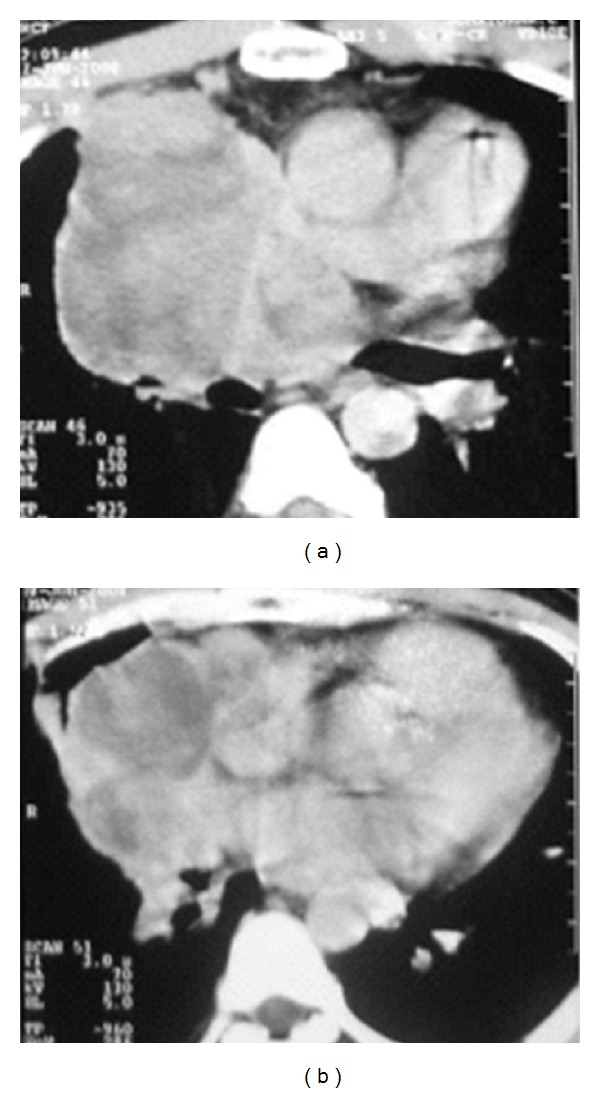
Axial post-contrast CT scan of the chest showing areas of necrosis in the mass with adherence to the pericardium.

A 42-year-old man presented with symptoms of right-sided chest pain, not related to physical activity or exertion. He also complained of shortness of breath on exertion. There was no history of cough, fever, or weight loss. Physical examination did not reveal anything significant. His haematological parameters were normal. The chest radiograph (PA view) showed a well-defined radio opaque shadow with no calcification or cavitations with its medial border merging with the right border of the heart, which was suggestive of an anterior mediastinal mass (Figure 1(a)). Lateral view confirmed the anterior mediastinal location of the mass (Figure 1(b)). An axial CT scan showed a heterogeneously enhancing right anterior mediastinal mass with areas of necrosis which was adherent to the pericardium (Figure 2). Bronchoscopic examination showed extrinsic compression of the trachea and the right main bronchus. With a provisional clinical diagnosis of a lymphoma versus a teratoma, surgical excision was planned through an anterolateral approach. Per operatively, a 6 × 7 cm mass was found in the anterior mediastinum which had a smooth surface. The mass showed plenty of adhesions to the right lung and pleura. The mass was removed in piecemeal and was sent with a portion of the lung for histopathological evaluation. 

**Figure 3:**
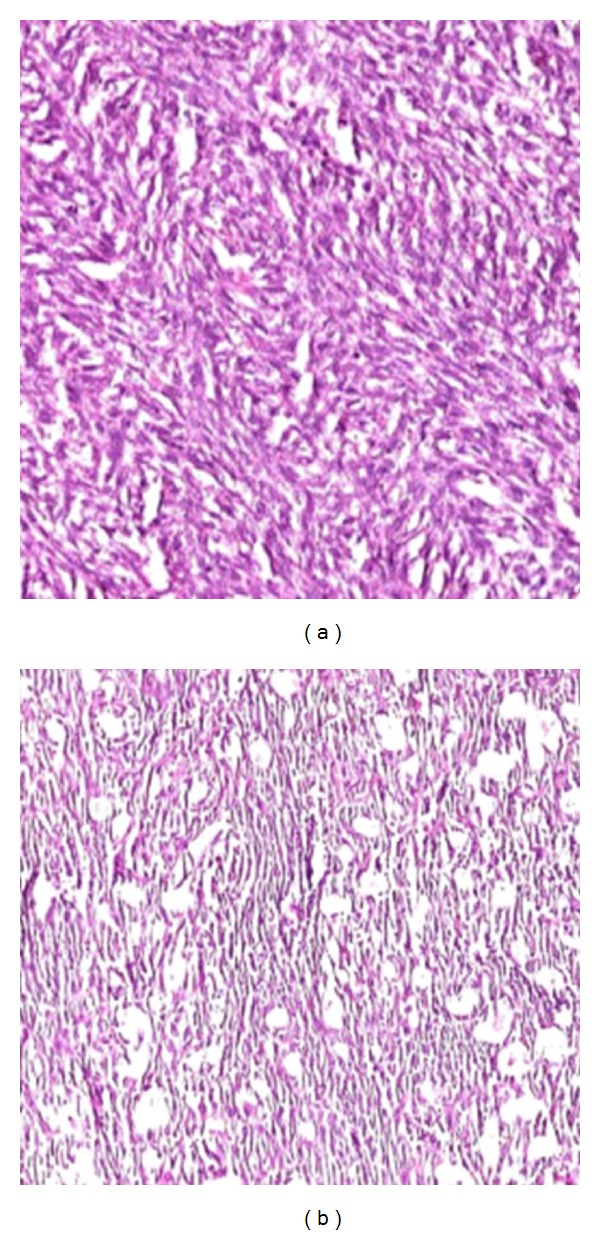
Photomicrographs showing the spindle cell (a) and the epithelial components (b) of the neoplasm (×100, H&E).

**Figure 4:**
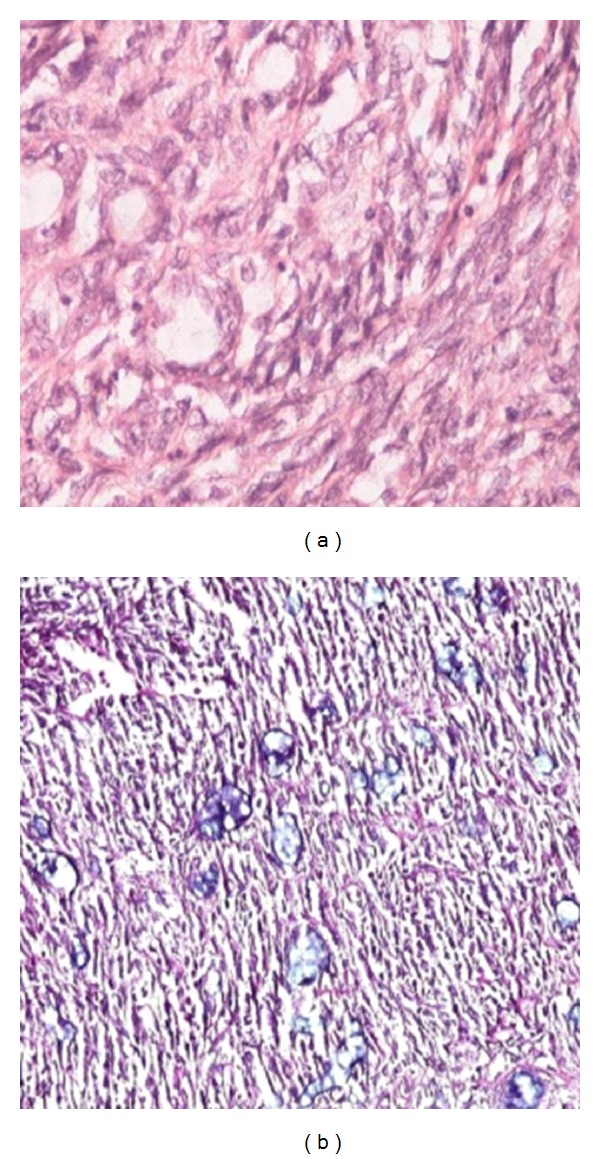
Photomicrographs highlighting the glandular features of the tumour (a) with Alcian blue positivity in the lumen (b) (×400, H&E; ×100, Alcian blue, resp.).

 The gross specimen consisted of about 250 gm of grey white soft tissue with extensive areas of necrosis. Also received was a lobe of lung measuring 9 × 9 × 2 cm. On microscopic examination, a biphasic neoplasm was noted with well formed glandular epithelial structures admixed with a spindle cell component (Figure 3). The spindle cell component was monomorphous and showed a fascicular arrangement. The cells showed elongated vesicular nuclei with mild to moderate nuclear atypia. Mitosis ranged from 3–5 per 10 high power fields. The glandular spaces were lined by cuboidal cells with vesicular nuclei and moderate cytoplasm and showed eosinophilic secretions within their lumens which stained positively with the Alcian blue stain (Figure 4). The toluidine blue stain failed to reveal any mast cells. There were foci of calcification and also large areas of necrosis and haemorrhage. The lung parenchyma and pleura showed no evidence of infiltration by tumour. 

**Figure 5:**
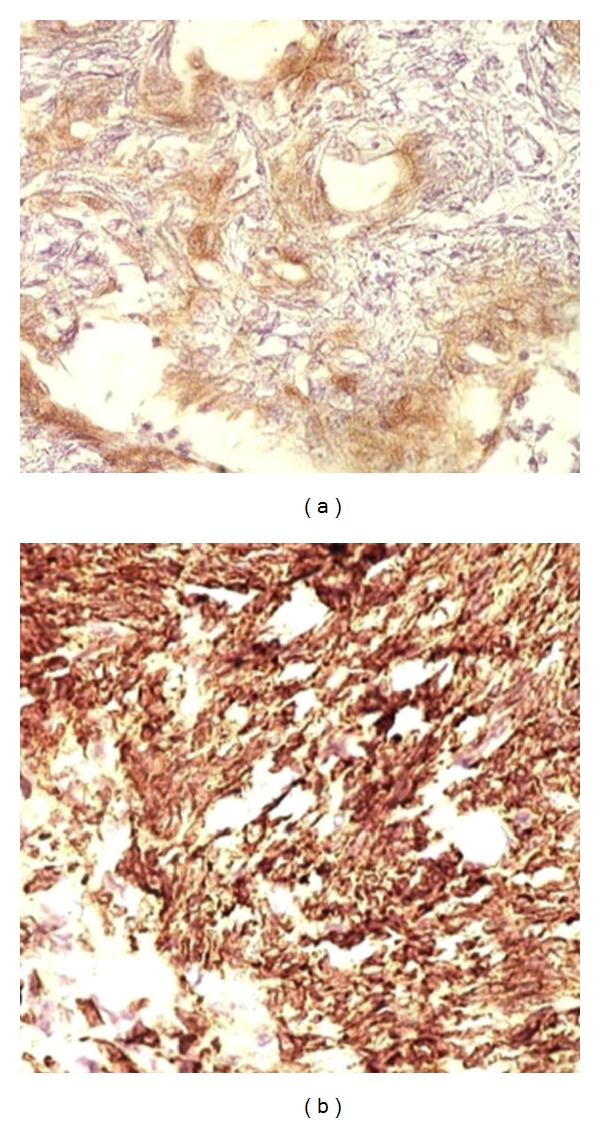
Immunohistochemistry showing positivity for EMA (a) and Bcl-2 (b) (×400 and ×200, resp.).

 On immunohistochemistry, the glandular component showed cytoplasmic positivity for the epithelial markers cytokeratin and epithelial membrane antigen (EMA). The spindle cell component stained strongly with vimentin and bcl-2 (Figure 5). The tumour did not stain with leucocyte common antigen LCA, desmin, S100, smooth muscle actin (SMA), CD34, or CD99. The proliferation marker Ki67 showed nuclear positivity in up to 10% of the tumour cells. With the combination of histopathological features and immunohistochemical findings, a diagnosis of a primary synovial sarcoma of the mediastinum was offered. The patient received adjuvant chemotherapy with Ifosphamide and Doxorubicin. Eight months later, he presented again with distant metastasis. 

## 3. Discussion

Synovial sarcoma is a rare and aggressive malignant soft tissue tumour occurring most commonly in the extremities of young adults. Other sites from which this tumour has been reported include the lung, pleura, chest wall and very rarely, the mediastinum [2–6]. Only a few case reports, and series of primary mediastinal synovial sarcoma are described in the literature so far. A vast array of tumours occur in the mediastinum, both primary and secondary, and the diagnosis of a primary mediastinal synovial sarcoma can be challenging. 

Witkin et al. for the first time in 1989 reported 4 cases of biphasic synovial sarcomas of the mediastinum [4]. The patients were all adult males, and the tumours were frequently adherent to the adjacent pleura or pericardium but none were arising from them. Keratin positivity was seen in the epithelial elements and vimentin positivity in the spindle cell elements of these tumours. Adherence to the pleura was a feature noted in our case also. In addition to immunohistochemistry, molecular genetic analysis may be performed to demonstrate the t(x;18) translocation [6]. A series of 15 cases of primary synovial sarcomas were reported by Suster and Moran. They described the clinicopathological, immunohistochemical, and ultrastructural features of these tumours. Nine of their cases presented with anterior mediastinal masses with chest pain and shortness of breath, in a way similar to our case. The rest showed a posterior mediastinal location. Five of their cases showed a biphasic appearance, the rest being monophasic. Immunohistochemistry done on 10 cases showed focal positivity for cytokeratin and/or epithelial membrane antigen (EMA) and strong positivity for vimentin and bcl-2 in the spindle cells. Our case also showed strong positivity for vimentin and bcl-2 in the spindle cell areas with focal cytokeratin and EMA positivity in the glandular elements. 

The differential diagnosis of a primary synovial sarcoma in the mediastinum is complex as a wide array of primary and metastatic tumours occur in this site. The biphasic synovial sarcoma must be distinguished from metastatic sarcomatoid carcinoma and biphasic malignant mesothelioma. In a sarcomatoid carcinoma, the spindle cells as well as the epithelial elements show atypical features. The glandular structures in a synovial sarcoma do not exhibit much pleomorphism. Biphasic mesothelioma can show morphological features very similar to a biphasic synovial sarcoma. However grossly, a mesothelioma presents as diffuse plaque like tumour masses on the pleural or pericardial surfaces, whereas a synovial sarcoma presents as a discrete mass in the mediastinum. Our case presented as a discrete mass in the mediastinum and showed adhesions to the pleura. Other spindle cell tumours that commonly occur in the mediastinum like solitary fibrous tumour and malignant peripheral nerve sheath tumour (MPNST) were excluded in this case, as the tumour cells were negative for CD34 and S100 respectively. The gross findings in combination with the morphological and immunohistochemical profile confirmed the diagnosis of a synovial sarcoma in our case. 

Bcl-2 is the product of a mitochondrial oncogene which inhibits apoptosis. This oncoprotein is found to be expressed in a variety of benign and malignant spindle cell tumours of the skin and soft tissue [7]. Its greatest diagnostic usefulness, however, seems to be in synovial sarcoma and solitary fibrous tumour. However, the latter also shows positivity for CD34 which is not seen in synovial sarcoma. 

Synovial sarcomas are highly aggressive tumours, and complete surgical excision remains the mainstay of treatment. Radiotherapy is often required to obtain local control of the disease with adjuvant chemotherapy. Adequate pre operative work up such as a fine needle aspirate (FNA) or a needle biopsy might have offered a clue to the diagnosis in this case perhaps leading to better preoperative planning and *en bloc* resection of the mass.

 In conclusion, synovial sarcoma must be entertained in the differential diagnosis of spindle cell as well as biphasic tumours arising in the mediastinum. This case report emphasizes the importance of prompt clinical suspicion, accurate histopathological diagnosis, and use of appropriate immunohistochemical markers in the diagnosis of this unusual tumour in an unusual site.
